# Psychometric Validation Study of the Liebowitz Social Anxiety Scale - Self-Reported Version for Brazilian Portuguese

**DOI:** 10.1371/journal.pone.0070235

**Published:** 2013-07-26

**Authors:** Larissa Forni dos Santos, Sonia Regina Loureiro, José Alexandre de Souza Crippa, Flávia de Lima Osório

**Affiliations:** 1 Department of Neurosciences and Behavior, Faculty of Medicine of Ribeirão Preto – USP, Ribeirão Preto, Sao Paulo, Brazil; 2 National Institute of Technology and Translational Medicine, Ribeirão Preto, Sao Paulo, Brazil; Institute of Psychiatry at the Federal University of Rio de Janeiro, Brazil

## Abstract

Social Anxiety Disorder (SAD) is prevalent and rarely diagnosed due to the difficulty in recognizing its symptoms as belonging to a disorder. Therefore, the evaluation/screening scales are of great importance for its detection, with the most used being the Liebowitz Social Anxiety Scale (LSAS). Thus, this study proposed to evaluate the psychometric properties of internal consistency and convergent validity, as well as the confirmatory factorial analysis and reliability of the self-reported version of the LSAS (LSAS-SR), translated into Brazilian Portuguese, in a sample of the general population (N = 413) and in a SAD clinical sample (N = 252). The convergent validity with specific scales for the evaluation of SAD and a general anxiety scale presented correlations ranging from 0.21 to 0.84. The confirmatory factorial analysis did not replicate the previously indicated findings of the literature, with the difficulty being in obtaining a consensus factorial structure common to the diverse cultures in which the instrument was studied. The LSAS-SR presented excellent internal consistency (α = 0.90–0.96) and test-retest reliability (Intraclass Correlation Coefficient = 0.81; Pearson’s = 0.82). The present findings support those of international studies that attest to the excellent psychometric properties of the LSAS-SR, endorsing its status as the gold standard.

## Introduction

Social Anxiety Disorder (SAD) is characterized by marked and persistent fear of social situations, leading the individual to phobic avoidance behavior, which can result in a major impact in the life quotidian [1]. Despite its high prevalence, it is a rarely diagnosed disorder [2], as the symptoms can often be confused with personal characteristics, especially in certain cultures. Therefore, evaluation/screening instruments are very important in helping healthcare professionals to screen for and correctly diagnosis SAD, so that a specific therapeutic approach can be defined, supporting the sufferer in adequately coping with social activities. The Liebowitz Social Anxiety Scale (LSAS) is the most studied scale regarding its psychometric proprieties, when compared to the other scales available for measuring SAD symptoms [3], with it also being the most used scale in clinical studies [4]–[7]. A literature review concerning the treatment of SAD in adults, showed that the LSAS is the most used instrument to access SAD symptomatology, as it was used in around 90% of the randomized clinical trials performed from 2005 to 2010 [8].

The LSAS was developed in 1987, by Michael Liebowitz [9], and is composed of 24 items, scored on a Likert-type scale which varies between 0 and 3 points (none/never - severe/usually), related to the fear and avoidance of different social situations experienced in the previous week. It has already been translated, adapted and validated into four languages in addition to the original English, these being French [10], Spanish [11], Hebrew [12] and Turkish [13]. All these versions presented excellent psychometric indicators.

It is important to highlight that initially, the LSAS was proposed as a clinician-administrated scale, however, later studies [14]–[16] have considered the use of the self-reported version, since it presents psychometric qualities as satisfactory as the clinician-administrated version [17].

Regarding the studies which aimed to analyze the psychometric proprieties of the self-reported version of the LSAS (LSAS-SR), all of them found good results when evaluating the internal consistency parameter, with the *alpha* values varying between 0.61 and 0.98 [14], [17], [18].

In relation to the concurrent validity, the instruments used to perform this correlation varied according to the study, however, the results always presented an acceptable fit (Social Phobia Scale: r = 0.44–0.80; Social Interaction Anxiety Scale: r = 0.33–0.80). For example, Backer et al. [14] found a moderate to excellent correlation using the Social Phobia Scale (0.44–0.80).

Regarding the divergent validity, the most common correlations were performed using scales that evaluate depression and general anxiety symptoms, in both cases the correlations were classified as weak to moderate. For example, two studies conduced with clinical samples in the USA [14], [16], using the Beck Depression Inventory, found similar correlations (0.25–0.46 [12]; 0.44–0.48 [14]).

The most contradictory point in the psychometric studies of the LSAS-SR is the factorial analyses [3]. When the scale was developed, Liebowitz [9] suggested a two-factor structure in which the subscales (fear and avoidance) represent the factors. It is important to remember that this first version of the scale was clinician-administrated. Subsequently, many groups proposed different structures, as the first one did not show such a good fit when using different samples and different LSAS versions, such as the self-report and the clinician-administrated versions. The most widespread structures are of three [12], four [19] or five factors [14], the first two used the clinician-administrated and the last the self-reported version. A possible reason for this impasse is the cultural differences in the samples used, as it is known that SAD symptoms can be rated in different ways according to the cultural context [3].

Considering that no other research, to our knowledge, has been performed to date with the LSAS-SR in its adaptation to Brazilian Portuguese, the study of its psychometric characteristics, i.e., convergent validity, confirmatory factorial analysis, internal consistency and test-retest reliability, was proposed.

## Materials and Methods

### Ethics Statement

This study was approved by the Human Research Ethics Committee of the Clinical Hospital of Ribeirao Preto Medical School- Sao Paulo University (11570/2003-HCRP). During the data collection, all guidelines regarding the ethical considerations in research with humans [20] were adhered to, so that only those subjects that agreed to participate, by signing the Terms of Free Prior Informed Consent (TFPIC), were included. This form was given to the subjects individually, with children and adolescents below 18 years of age being excluded from the study. The subjects that were identified with SAD were offered information and treatment.

### Study Characterization

The study was conducted in two phases, with different samples and aims. The first involved the participation of 269 subjects and aimed to perform the study of the reliability through the test-retest technique. The second, composed of 252 subjects, aimed to perform the study of the convergent validity, confirmatory factorial analysis and internal consistency.

### Instruments

#### The Liebowitz Social Anxiety Scale self-reported version (LSAS-SR)

Developed by Liebowitz [9], consists of a scale which evaluates SAD symptoms related to the fear and avoidance of different social situations experienced in the previous week. It is divided into two subscales (fear and avoidance) and composed of 24 items, scored on a Likert-type scale of four points. Originally proposed as a clinician-administrated scale, the self-reported version may also be used. The clinician-administrated version translated by Lotufo-Neto [21] was transformed into the self-reported version [22];

#### The Social Phobia Inventory (SPIN)

Proposed by Connor et al. [23] aims to access the intensity of physiological symptoms, fear, and avoidance in different anxiogenic situations related to SAD, experienced in the previous week. It was translated and adapted into Brazilian Portuguese by Osório et al. [24], [25]. This self applied instrument is composed of 17 items, divided into three subscales (fear, avoidance and physiological symptoms), scored on a Likert-type scale of zero (none) to four (extremely), and is used to quantify the physiological symptoms, fear and avoidance associated with SAD;

#### The Mini-SPIN

Was proposed by Connor et al. [26] and translated and adapted into Brazilian Portuguese by Osório, et al. [27], [28]. This is a reduced instrument, consisting of three of the 17 items of the SPIN (items 6, 9, 15), which proved, in the psychometric study, to be the most discriminative for subjects with SAD;

#### The Brief Social Phobia Scale (BSPS)

Was proposed by Davidson et al. [29] and translated and adapted into Brazilian Portuguese of by Osório et al. [30], [31]. This clinician-administered scale is composed of 18 items, on three subscales (fear, avoidance and physiological symptoms) that consider the different characteristic symptoms of SAD experienced in the previous week and is scored on a five-point Likert-type scale (0 = none/never; 4 = extreme/always);

#### The Beck Anxiety Inventory (BAI)

Was proposed by Beck et al. [32] and translated, adapted and validated for Brazilian Portuguese by Cunha [33]. This self-administered instrument is composed of 21 items that evaluate the intensity of general anxiety symptoms experienced in the previous week and is scored on a Likert-type scale of five points (0 = not at all; 4 = severely). It is divided into four subscales: neurophysiological, subjective, panic and autonomic;

#### The Structured Clinical Interview for the DSM-IV - diagnostic and statistical manual of mental disorders, 4^th^ ed

Was proposed by First et al. [34] and translated and adapted into Portuguese by Del-Ben et al. [35]. This instrument consists of an interview script, composed of ten modules, used for the development of psychiatric clinical diagnoses based on the DSM-IV. It should be noted that Module F refers to Social Anxiety;

#### Identification questionnaire

Composed of 16 items directed towards the sociodemographic characterization of the subjects.

### Sample Selection and Data Collection Procedure

For the realization of both phases of the study, contact was made with the coordinators of the universities and their authorization obtained to carry out the study. Courses and disciplines with the highest number of students enrolled were chosen by convenience and then the professor was asked for authorization to perform the data collection in the classroom, where the study aims were explained to the students. Those who agreed to participate were given the TFPIC and after their formal acceptance they were given an application notebook containing the following instruments: LSAS-SR, SPIN/Mini-SPIN and Identification Questionnaire.

For Phase 1 of the study, the data collection was conducted in two stages, with an interval of 15 days, in order to check the reliability through the test-retest technique. The following inclusion criteria were used: individuals of both sexes, aged over 18, who agreed to participate in the study by signing the TFPIC. The exclusion criteria were: less than 18 years of age and incorrect completion of the instruments. Thus, in the two days of the application a total of 413 of the subjects contacted agreed to participate. Of these, 128 were not present on the first day of the application and another 16 absent on the second day. Therefore, these subjects (N = 144) were excluded from the reliability study of the LSAS-SR, with the final sample composed of 269 subjects.

**Figure 1:**
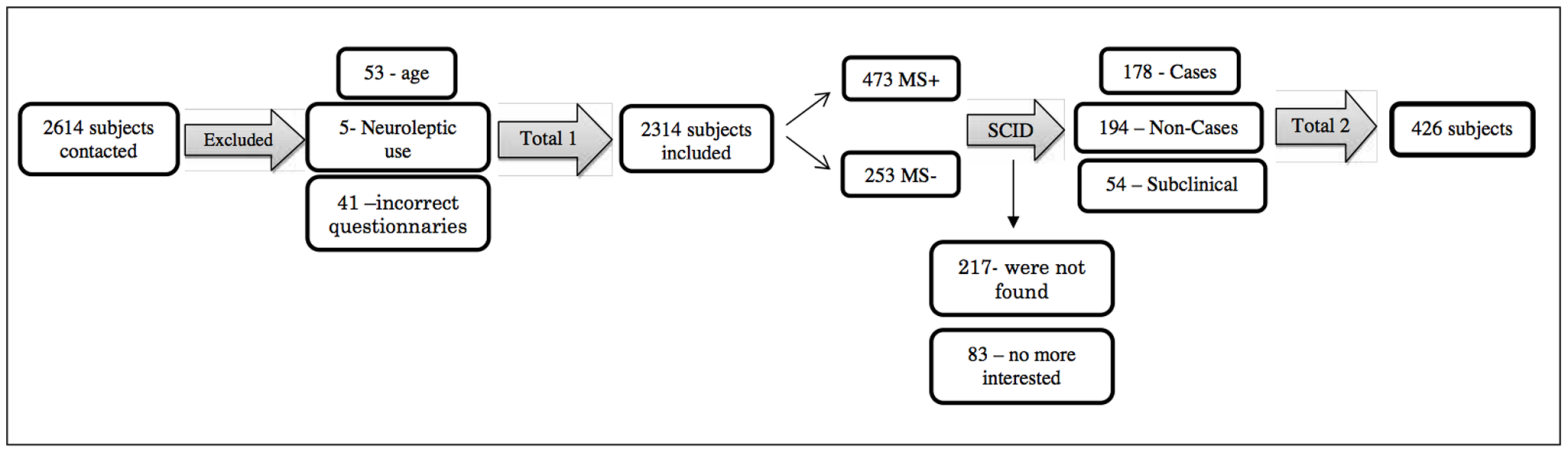
First steps of the Phase 2 sample path. MS+: Positive diagnoses of SAD according Mini-SPIN; MS–: Negative diagnoses of SAD according Mini-SPIN.

Phase 2 included subjects of both sexes, aged between 18 and 35 years, who agreed to participate in the study by signing the TFPIC. The exclusion criteria were: use of neuroleptics, presence of the following psychiatric comorbidities: psychotic manifestations, current depression, recurrent depression, current eating disorder, obsessive compulsive disorder, hypomanic/manic episodes or panic disorder, as well as the incorrect completion of the instruments. Figure 1 presents the first steps of the data collection.

As can be seen in Figure 1, a total of 2614 students were contacted for the initial screening for potential SAD cases. Of these, 201 refused to participate in the study, citing lack of interest or availability, 53 were excluded for being aged below 18 or over 35 years, 41 for incorrect completion of the instruments and five due to the use of neuroleptics, giving a final sample of 2314 subjects.

From this sample, all the subjects (N = 473) who scored greater than or equal to 6 in the Mini-SPIN (Mini-SPIN Positive) were selected as the clinical group, while 253 other subjects, with Mini SPIN scores less than or equal to 2 (Mini-SPIN Negative - approximately half the total of the Mini-SPIN Positive group), were randomly selected to compose the comparison group.

All the subjects were contacted by telephone to answer Module F of the SCID-IV. It was not possible to make contact with 217 of these and 83 claimed to have no more interest in participating. The remaining subjects (N = 426) were divided into three groups according to the following inclusion criteria:

#### Case group (C)

Score greater than or equal to six in the Mini-SPIN (Mini-SPIN Positive), according to the criteria of Connor et al. (2001) and module F of the SCID-IV positive (N = 178);

#### Non-case group (NC)

Score less than or equal to two in the Mini-SPIN (Mini-SPIN Negative) and module F of the SCID-IV negative (N = 194);

#### Subclinical group (SC)

Scores greater than or equal to six in the Mini-SPIN (Mini-SPIN Positive), according to the criteria of Connor et al. (2001) and positive for all the criteria of module F of the SCID-IV, except for criterion E, regarding the presence of suffering and social impairment (N = 54).

Subsequently, these subjects were contacted again and invited to attend the Psychopharmacology Laboratory of the Clinical Hospital of the Faculty of Medicine of Ribeirão Preto-USP, to participate in an interview, in order to apply the SCID-IV and a broad battery of SAD evaluation instruments. Table 1 presents the composition of the final sample.

**Table 1:** Contacted, included and excluded subjects in the different groups of the study Phase 2.

Subject situation	C	NC	SC	Total
Selected	178	194	54	426
Excluded:				
- Quit or not located	40	92	15	147
- Comorbidities	18	5	0	23
- Form incorrectly completed	2	2	0	4
**Included**	**118**	**95**	**39**	**252**

C: Cases; NC: Non-cases; SC: Subclinical cases.

According to Table 1, after applying the exclusion criteria, the final sample was composed of 118 SAD cases, 95 non-cases and 39 subclinical cases, giving a total of 252 subjects.

### Data Analysis

The data were manually encoded and stored in a database. The SPSS version 13.0 [36] program was used for the analyzes, with Mplus version 6.12 [37] used to perform the confirmatory factorial analysis. The demographic and clinical data of the sample were analyzed using descriptive and parametric statistical tests. To compare the groups the chi-square test and ANOVA were used.

For the studies concerning the validity of the LSAS the following analytical techniques were used:

Cronbach’s alpha, in order to evaluate the internal consistency of the scale. Values exceeding 0.60 [38] were considered acceptable;
Pearson’s Correlation Coefficient (r) for the items, total scores and for the subscales of the LSAS-SR, SPIN, BSBP and BAI. To classify the magnitude of the correlations, the following standards were used: 0 to 0.25: weak; 0.26 to 0.50: moderate; 0.51 to 0.70: strong; and above 0.71 very strong [39], [40];
Kappa Correlation Coefficient, Intraclass Correlation Coefficient and Pearson’s Correlation Coefficient - to evaluate the reliability of the scale using the test-retest technique – with confidence intervals of 95% adopted as the level of significance. The parameters for the classification of the correlations were the same as mentioned above [39], [40];
Confirmatory factorial analysis was performed, using the factors highlighted in the studies of Levin et al. [12] Safren et al. [19] and Baker et al. [14], as described below:
  - Levin et al. [12] (three factors, considering only the fear subscale): Factor 1 – Group Performance and Interaction (items 2, 6, 7, 14, 15, 16, 20, 23); Factor 2 – Dyadic Interaction (items 5, 10, 11, 12, 18, 19, 21, 22, 24); Factor 3 – Public Activities (items 1, 3, 4, 8, 9, 13, 17);
  - Safren et al. [19] (four factors, considering only the fear subscale): Factor 1 – Social Interaction (items 5, 7, 10, 11, 12, 19, 21, 18); Factor 2 – Public Speaking (items 2, 6, 15, 16, 20); Factor 3 – Observation (items 1, 9, 13, 17); Factor 4 – Eating and Drinking in Public (items 3, 4);
  - Baker et al. [14] (five factors, considering the fear and avoidance subscales): Factor 1 – Social Interaction Anxiety (items 5, 7, 10, 11, 12, 14, 18, 19, 21); Factor 2 – Non-verbal Performance Anxiety (items 8, 9, 17); Factor 3 – Ingestion Anxiety (items 3, 4); Factor 4 – Public Performance Anxiety (items 6, 15, 16, 20); Factor 5 – Assertiveness Anxiety (items 1, 13, 22, 24).

Analysis was carried out using the Weighted Least Squares (WLS) method, with the estimate being established through the mean and the covariance adjusted according to the chi-square test. The following parameters were adopted as goodness-of-fit model indicators: root-mean square error of approximation (RMSEA), comparative fit index (CFI), and Tucker-Lewis Index (TLI). As recommended by Hu and Bentler [41], [42], a excellent fit is considered when RMSEA ≤0.06 and, CFI and TLI ≥0.95.

The Student t-test and ANOVA were used to compare the variables, using the Bonferroni post-hoc test. The significance level adopted was p≤0.05.

## Results

### Sociodemographic Characterization of the Samples

To carry out this study, as previously stated, two different samples were used.

The main sociodemographic characteristics of the samples included in both phases of the study can be seen in Table 2.

**Table 2:** Sociodemographic characterization of the sample included in Phase 2 of the study (N = 252).

Variable	C (N = 118) N (%)	NC (N = 95) N (%)	SC (N = 39) N (%)	Statistic (C*x*NC*x*SC)	GP (N = 269) N(%)
**Gender**					
Female	81(68.6)	56 (58.9)	30 (76.9)	χ^2^ = 4.789	200 (74.0)
Male	37 (31.4)	39 (41.1)	9 (23.1)	p = 0.91	69 (26.0)
**Mean age (SD)**	22.63 (5.39)*	20.93 (2.86)*	21.29 (3.1)	F = 4.150 p = 0.017*	23.40 (6.22)
**Field of study**					
Exact Sciences	37 (31.4)	33 (34.7)	13 (33.3)	χ^2^ = 0.327	–
Humanities	14 (11.9)	11 (11.6)	5 (12.8)	p = 0.988	–
Biological Sciences	67 (56.7)	51 (53.7)	21 (53.9)		269 (100%)
**Year of course**					
1^st^ and 2^nd^	75 (63.6)	70 (73.7)	30 (76.9)	χ^2^ = 3.759	214 (79.6)
3^rd^ and 4^th^	43 (36.4)	25 (26.3)	9 (23.1)	p = 0.153	55 (20.4)

C: Cases; NC: Non-Cases; SC: Subclinical cases; N: Frequency; %: Percentage; SD: Standard Deviation; χ^2^: Chi-squared test; F = ANOVA; *Statistically significant difference.

According to Table 2, the first sample group (Phase 1) was composed of 269 university student subjects with a mean age of 23.4 years (SD = 6.22), the majority of whom were female (74.0%), studying the biological sciences, and enrolled in the first two years of the course (79.6%).

For the clinical sample (Phase 2), all the groups were predominantly composed of female students, enrolled in the first years of the university course, mostly in the biological sciences. In the statistical analysis of these variables a small difference was found between the ages of the subjects that comprised the C and NC groups, i.e., in group C the mean age was higher than the mean age of the NC group, both of which did not differ, from a statistical point of view, from the mean age of the SC group.

### Convergent Validity

Regarding the convergent validity study, the scores of the subscales of the LSAS-SR, as well as its total score, were correlated with the scores of the subscales and totals of the following evaluation instruments: SPIN, BSPS and BAI. These correlations were performed only with the clinical samples, with the results presented in Table 3.

**Table 3:** Values relative to the study of the convergent validity of the Liebowitz Social Anxiety Scale - self-reported version (LSAS-SR) and its subscales with the Social Phobia Inventory (SPIN), Brief Social Phobia Scale (BSPS) and Beck Anxiety Inventory (BAI), in a sample of the general population (N = 413) and in a clinical sample (N = 252).

	LSAS-SR								
	Cases (N = 118)			Non-Cases (N = 95)			Subclinical Cases (N = 39)		
	FS	AS	Total	FS	AS	Total	FS	AS	Total
***SPIN***									
**FS**	0.61**	0.61**	0.63**	0.76**	0.77**	0.80**	0.41**	0.33*	0.39*
**AS**	0.61**	0.64**	0.64**	0.68**	0.67**	0.71**	0.40*	0.39*	0.41*
**PSS**	0.52**	0.51**	0.53**	0.66**	0.70**	0.71**	−0.01	−0.05	−0.03
**Total**	0.65**	0.66**	0.67**	0.77**	0.78**	0.81**	0.34*	0.29	0.33*
***BSPS***									
**FS**	0.71**	0.66**	0.70**	0.76**	0.69**	0.76**	0.76**	0.67**	0.75**
**AS**	0.68**	0.73**	0.73**	0.77**	0.77**	0.81**	0.72**	0.74**	0.77**
**PSS**	0.55**	0.51**	0.54**	0.58**	0.49**	0.56**	0.19	0.12	0.16
**Total**	0.75**	0.73**	0.76**	0.82**	0.77**	0.84**	0.76**	0.70**	0.77**
***BAI***									
**NS**	0.21*	0.23*	0.23*	0.51**	0.42**	0.49**	−0.09	−0.09	−0.09
**SS**	0.37**	0.34**	0.37**	0.41**	0.46**	0.46**	−0.05	−0.07	−0.06
**PS**	0.34**	0.34**	0.35**	0.29**	0.29**	0.30**	−0.01	−0.15	−0.09
**AuS**	0.36**	0.35**	0.37**	0.49**	0.39**	0.46**	0.24	0.13	−0.19
**Total**	0.37**	0.36**	0.38**	0.55**	0.52**	0.57**	−0.01	−0.06	−0.03

FS: Fear Subscale; AS: Avoidance Subscale; PSS: Physiological Symptoms Subscale; NS: Neurophysiological Subscale; SS: Subjective Subscale; PS: Panic Subscale; AuS: Autonomic Subscale;

* p≤0.05; **p≤0.01.

Taking the SPIN as the reference, in both the C group and the NC group, all the correlations found were statistically significant and classified as strong to very strong (r = 0.52–0.81), with them being slightly lower in the C group than the NC group. In the SC group the standard of correlation with SPIN was statistically lower than in the other groups, being generally classified as moderate (r = 0∶33–0∶41), with no significant correlations presented with the physiological symptoms subscale.

Regarding the BSPS, a very similar standard of correlation was found between the different clinical groups, being classified as strong to very strong (r = 0.67–0.84). However, with respect to the correlations with the physiological symptoms subscale of the BSPS, this standard was somewhat lower, with none of the correlations with this subscale being statistically significant for the SC group.

The correlations performed between the LSAS-SR and BAI tended not to present a specific standard, ranging from 0.21 to 0.57, being classified as weak to strong, in the C and NC groups. For the SC group no statistically significant correlation was observed.

### Confirmatory Factorial Analysis

Confirmatory factorial analysis was performed by testing different models previously described in the literature [12], [14], [19], as can be seen in Table 4.

**Table 4:** Indicators of factorial model goodness-of-fit according to the Weighted Least Squares (WLS) method.

Model	χ^2^	df	CFI	TLI	RMSEA	RMSEA 90% CI
**Levin et al. (2002) 3 factors** [**12**]	9595.350	1081	0.842	0.834	0.102	0.097–0.108
**Safren et al. (1999) 4 factors - AS** [**39**]	1985.079	153	0.919	0.904	0.099	0.083–0.115
**Safren et al. (1999) 4 factors - FS** [**39**]	2161.201	153	0.951	0.942	0.081	0.063–0.098
**Baker et al. (2002) 5 factors** [**14**]	2688.748	231	0.957	0.950	0.067	0.051–0.082

FS: Fear Subscale; AS: Avoidance Subscale; χ^2^: chi-square; df: degree of freedom; CFI: Comparative Fit Index; TLI: Tucker-Lewis Index; RMSEA: Root Mean Square Error of Approximation; CI: Confidence Interval.

Given the pre-defined parameters, none of the models were considered to be an extremely good fit for the sample of the present study. That of Baker et al. [14] was the closest to such a fit, with the parameters within expectations regarding the CFI and TLI, however, with the RMSEA slightly varying from the proposed pattern, which does not allow the model to be accepted.

### Reliability

#### Internal consistency

Regardless of the sample, the alpha value was excellent, for the fear subscale the values found were between 0.91 (SC) and 0.93 (C), for the avoidance subscale 0.90 (NC) to 0.92 (C) and for the total score, 0.95 (NC and SC) to 0.96 (C). An analysis of internal consistency was also conducted simulating the removal of each of the items of the instrument and it was observed that it did not need to be altered, with no difference regarding the group in question.

#### Test-retest

Aiming to evaluate the temporal stability of the LSAS-SR, the ICC was calculated for the total scale, with a value of 0.81 obtained (CI: 0.77–0.85), classified as excellent. This same standard was found for the fear subscale score (0.81; CI: 0.76–0.85). Regarding the avoidance subscale scores, there was a slight decrease in the ICC value (0.77; CI: 0.72–0.82).

The Pearson’s correlation between the total scores at both moments of evaluation was also calculated. The values found were classified as excellent for the total score (r = 0.82) and for the subscales of fear (r = 0.82) and avoidance (r = 0.78). Furthermore, the evaluation of the temporal stability was performed for each item individually, through the kappa, ICC and Pearson’s tests. For the items of the fear subscale, the ICC values ranged from 0.42 (item M4) to 0.68 (items M6 and M16), the kappa indices from 0.29 (item M12) to 0.44 (item M6) and the Pearson’s correlation index from 0.42 (item 4) to 0.70 (item M6). Regarding the avoidance subscale, the lowest ICC was 0.34 (item E12) and the highest 0.68 (Item E6), while the kappa values ranged from 0.26 (item E18) to 0.47 (item E7) and the Pearson correlation index from 0.36 (item E12) to 0.85 (item E24).

## Discussion

The final sample was composed of four separate groups of subjects, involving the general population, cases, non-cases and subclinical cases of SAD, however it presented limitation as it was composed only by university students from a specific region in Brazil, therefore the direct generalization of the findings need to be performed with caution. Regarding the sociodemographic characteristics of the samples, their homogeneity was evidenced in almost all the evaluated parameters, except age and university of origin, where small differences were found. However, it is believed that these aspects did not influence the results, with the homogeneity of the samples prevailing.

To perform the convergent validity study of the LSAS-SR, the SPIN and the BSPS were used primarily, with very significant correlation values expected, since they are all instruments that measure specific signs and symptoms of SAD. The SPIN and BSPS differ from the LSAS-SR in that they both have a subscale that evaluates the physiological symptoms associated with the disorder, with the BSPS, also being clinician-administered. The results encountered were in accordance with the initial hypothesis, as the correlations were classified as very strong.

It should be noted that the correlations between the subscales and total score of the LSAS-SR were significant and expressive, even when the physiological symptoms subscales of the SPIN (0.51–0.71) and the BSPS (0.49–0.58) are considered separately. Thus, it is clear that although the LSAS-SR does not posses items that evaluate this component of SAD, it was shown to be sensitive for a comprehensive evaluation of the social anxiety construct, especially in the C and NC groups.

Previous studies used different instruments to calculate the convergent validity. The two scales more commonly used for this purpose were the Social Phobia Scale (SPS) which evaluates the anticipatory anxiety and performance when being observed and the Social Interaction Anxiety Scale (SIAS), which evaluates behavior and cognition. The convergent validity indicators found were close to those of the present study, ranging from moderate to very strong (0.33–0.80) [14], [16], [19], [43]. When the BAI was used as the gold standard the correlation values found were weak to moderate. This result can be explained by the fact that the BAI is an instrument for evaluating general anxiety, while the LSAS-SR measures specific signs and symptoms of social anxiety. Again, the SC group presented a distinct standard of results, a possible reason for this could be the heterogeneity and specificity of the characteristic symptoms of this group.

A previous study [13], using a clinical sample, also correlated the BAI with the LSAS-SR, presenting similar results to the present study, although with a even lower standard (0.21–0.26). These low correlation indices also indicate the divergent validity between the instruments, reinforcing the need to use specific scales for the precise evaluation of SAD.

The factorial structure of the LSAS-SR does not present a model that achieves consensus among the researchers of the area, with different factorial solutions being found in the literature.

The confirmatory factor analysis was performed using the three factorial models previously proposed as the references, composed of three [12], four [19] or five factors [14]. None of the models fitted adequately for the study population. The five factor model proposed by Baker et al. [14] was the one with the parameters closest to the ideal, showing good incremental fit indices (TLI, CFI), but an inadequate parsimony fit index (RMSEA), preventing the use of this as a model for this sample.

The non-complete replication of the existing models can be explained considering two aspects: the sample composition and the statistical techniques used. Regarding the sample composition, the number of individuals that composed the study sample is highlighted, being slightly lower than that recommended. According to the literature, at least 150 to 300 subjects [44]–[46] should be used to perform the confirmatory factorial analysis, whereas in the present study the sample was 118. Regarding the statistical techniques employed, attention is drawn to the great diversity of techniques and parameters that have been previously used, which complicates the comparisons and, above all, the conclusion regarding the best factorial solution.

Considering the internal consistency, the values found in the present study were excellent, always above 0.90 for both the total score and for the subscales. These indices are in agreement with international studies that, regardless of the context and manner of application of the scale, always found high values for this parameter, ranging from 0.61 to 0.98 [11]–[14], [16]–[18], [42], [47]–[49].

Finally, the reliability analyzed through the test-retest technique presented excellent indicators, regardless of the statistical technique used, highlighting the good temporal stability and reproducibility of the LSAS-SR, which are in agreement with the international studies. In these, the indices encountered were considered good, varying from 0.78 to 0.97 [12], [13], [15].

Although the present study provides important findings regarding the LSAS-SR psychometric proprieties, it is necessary to highlight the particularity of the sample as a limitation, which, although it included a significant number of subjects, was composed only of university students. Therefore, caution should be taken in the generalization of the data for people of other sociocultural levels. This limitation does not, however, appear to have significantly interfered with the performance of the study and its results.

Generally, the validation of the LSAS-SR for the Brazilian context is important both for the development of research, as well as for screening in the clinical setting, considering that this is the most internationally used scale.

### Conclusions

It can be concluded that the initial aims of the study were achieved, especially considering that the validity and reliability of the LSAS-SR were measured through rigorous and extensive methodological analysis, even though the confirmatory factorial analyzes failed to find a good model fit. The importance of conducting cross-cultural validation studies should be emphasized, since these allow the adequation of the evaluation parameters of each instrument for a particular sociocultural context, which, until now, had not been conducted with the LSAS-SR in Brazil. The results of this study provide the instrument, which is the global reference for the evaluation of SAD, with the adequacy for use in Brazil and the psychometric findings presented attach more credibility to its use, both as a methodological resource in research, and as a screening tool in the clinical context.
